# Plasma exchange treats severe intrahepatic cholestasis caused by dacomitinib: A case report

**DOI:** 10.1097/MD.0000000000029629

**Published:** 2022-07-08

**Authors:** Fei Qiao, Qinlei Chen, Weiting Lu, Nanyuan Fang

**Affiliations:** a Department of Hepatology, Affiliated Hospital of Nanjing University of Chinese Medicine, Jiangsu Province Hospital of Chinese Medicine, Nanjing, China.

**Keywords:** ATP-binding cassette subfamily B member 1, case report, CYP2D6, dacomitinib, drug-induced liver injury, intrahepatic cholestasis

## Abstract

**Rationale:**

Dacomitinib-induced liver injury is often manifested by mild elevations of transaminases and bilirubin, and severe intrahepatic cholestasis caused by dacomitinib for simultaneous taking orally cytochrome P450 2D6 (CYP2D6) competitive substrates has been rarely reported.

**Patient concerns:**

The patient was a 69-year-old woman with non–small cell lung cancer (NSCLC) who was prescribed oral dacomitinib for a month; she was given oral loratadine due to “allergic rhinitis” and metoprolol extended action tablets due to “tachycardia” separately for a few days during the course of dacomitinib treatment. The patient developed liver damage, increased fatigue, yellow urine, and pruritus, with significantly elevated serum levels of bilirubin and glutamyltranspetidase.

**Diagnosis:**

Intrahepatic cholestasis, drug-induced liver injury, and NSCLC.

**Interventions:**

After admission, the patient was prescribed adenosylmethionine, acetylcysteine, ursodeoxycholic acid capsule, methylprednisolone and fenofibrate for a month, with progressive elevation of liver biochemical parameters. Through drug enzyme gene assays in the liver tissue after percutaneous liver biopsy, we found both CYP2D6*10/*10 and ATP-binding cassette subfamily B member 1 GG variants (rs1045642) positive. After the poor response to the conventional medication, the patient underwent plasma exchange.

**Outcomes:**

The patient was discharged after her liver parameters improved; the parameters remained normal at several follow-up visits, and she renewed the NSCLC regimens without dacomitinib after being evaluated by oncologists.

**Lessons:**

Dacomitinib can induce severe intrahepatic cholestasis. It is considered that patients with intermediate metabolic CYP2D6 are susceptible to drug-induced liver injury caused by dacomitinib; plasma exchange may be an effective treatment.

## 1. Introduction

Both the incidence and mortality rates of lung cancer rank the first among malignant tumors in China, and of which non–small cell lung cancer (NSCLC) accounts for about 85%. Most NSCLC patients are diagnosed at advanced stages with poor prognosis.^[1]^ The individualized molecular targeted therapy has become the base of treatment for advanced NSCLC due to its remarkable benefits and favorable safety profile. In particular, the development of epidermal growth factor receptor tyrosine kinase inhibitor (EGFR-TKI) is a hallmark in the evolution of therapies for NSCLC.^[2–4]^

Dacomitinib is one of second-generation, high-selective EGFR-TKI and widely used in clinical practice of NSCLC with EGFR mutants of exon 19 deletion or exon 21L858R substitution mutation. It irreversibly inhibits 3 different ErbB family molecule members, including EGFR/human epidermal growth factor receptor (HER) 1, HER2, and HER4.^[5]^ Dacomitinib-associated adverse events are often manifested by diarrhea, paronychia, dermatitis acneiform, interstitial lung disease, etc.^[6,7]^ Severe intrahepatic cholestasis, caused by dacomitinib from simultaneously taking cytochrome P450 2D6 (CYP2D6) competitive substrates, has been rarely reported.

## 2. Case report

**Figure 1.**
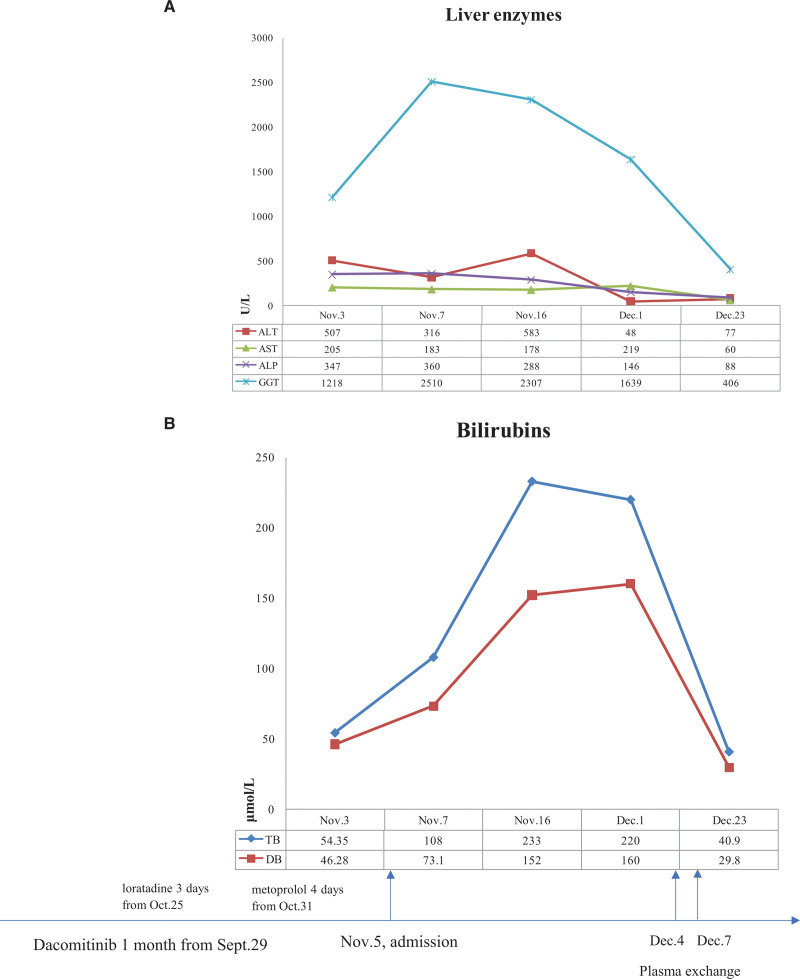
Parameters of liver enzymes and bilirubins at different time points in the course of the disease. (A) Liver enzymes, particularly relating to cholestasis, dramatically increased. (B) striking increased in total bilirubin, with direct bilirubin predominating. Plasma exchange was conducted on December 4 and December 7. ALP = alkaline phosphatase, ALT = alanine aminotransaminase, AST = aspartate aminotransaminase, DB = direct bilirubin, GGT = glutamyltranspetidase, TB = total bilirubin.

**Figure 2.**
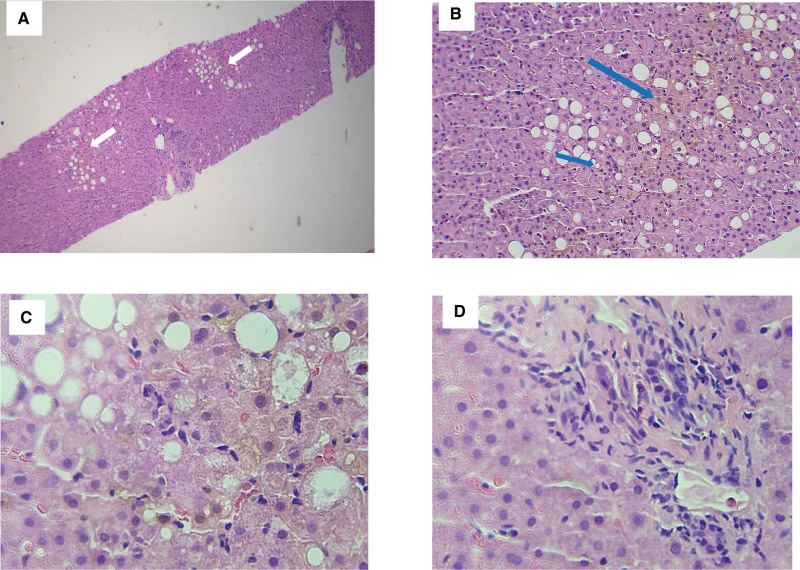
(A) 4X, Hematoxylin and eosin stain, liver biopsy histopathology showed hepatic steatosis (white arrow). (B) 20X, Hematoxylin and eosin stain, hepatocellular cholestasis (blue arrow) in acinus 3 zone. (C) 40X, Hematoxylin and eosin stain, ballooning degeneration and lymphocytic inflammation in parenchyma, especially acinus 3 zone. (D) 40X, Hematoxylin and eosin stain, mild to moderate inflammation mixed with neutrophilic and eosinophils at the site of portal tracts.

A 69-year-old female patient with NSCLC began to take dacomitinib 45 mg orally per day due to relapse of lung cancer on September 29, 2020. After taking the medicine, the patient reported fatigue. On October 25, 2020, loratadine was used to treat “allergic rhinitis” for 3 days. On October 31, 2020, the patient developed “tachycardia” and was treated with extended action tablets of metoprolol for 4 days. On November 3, 2020, the patient’s fatigue aggravated, accompanied by yellow urine and pruritus. The clinic laboratory tests revealed an abnormal biochemical parameters, with aspartate aminotransaminase (AST) 205 U/L (normal range: <32 U/L), alanine aminotransaminase (ALT) 507 U/L (normal range: <32U/L), alkaline phosphatase (ALP) 347 U/L (normal range: 50–135 U/L), glutamyltranspetidase (GGT) 1218 U/L (normal range: 7–32 U/L), total bilirubin (TB) 54.35 μmol/L (normal range: 5.1–28 μmol/L), and direct bilirubin (DB) 46.28 μmol/L (normal range: <10 μmol/L). On November 5, from the outpatient department, she was admitted into the department of hepatology of our hospital. The chief complaints were fatigue for a month, accompanied by yellow urine and pruritus for 2 days. The patient reported no alcohol consumption and denied taking other medications or substance abuse; she reported no exposure to chemicals and hepatotoxic substances; there was no family history of liver disease either. The physical examination at the initial contact did not reveal any abnormalities but icteric sclera. While the aforementioned oral medications were discontinued immediately, S-adenosyl methionine, acetylcysteine, and ursodeoxycholic acid capsules were prescribed orally. On November 7, ALT 316 U/L, AST 183 U/L, ALP 360 U/L, GGT 2510 U/L, TB 108 μmol/L, and DB 73.1 μmol/L were observed (Fig. 1); hepatitis B surface antigen, hepatitis B e antigen, hepatitis B virus antibodies profile, and antibodies of hepatitis C virus, hepatitis A virus, and hepatitis E virus were all negative; Epstein–Barr virus antibodies profile and cytomegalovirus antibodies were negative either. Immunofluorescence antinucleus antibodies, antismooth muscle antibody, anti-liver/kidney microsome, and antimitochondrial antibody had not been found. The level of ceruloplasmin was normal. No signs of extrahepatic biliary obstruction were found on ultrasound or magnetic resonance imaging. Methylprednisolone 80 mg was given orally for the exacerbation of the disease. Percutaneous liver biopsy was performed and liver drug enzyme genes were tested. By November 16, the liver biochemical parameters deteriorated further; AST 178 U/L, ALT 583 U/L, ALP 288 U/L, GGT 2307 U/L, TB 233 μmol/L, and DB 152 μmol/L were displayed. The pathological findings of the liver suggested hepatocellular cholestasis with ballooning degeneration and lymphocytic inflammation in parenchyma (Fig. 2). The diagnosis of intrahepatic cholestasis was established. On November 14, methylprednisolone was discontinued since it made little difference; fenofibrate 0.2 g was given orally for the increasing of serum lipid level. The reports of gene test suggested that genetic variants of CYP2D6*10/*10, CYP3A5*1/*3 and ATP-binding cassette subfamily B member 1 GG (rs1045642) were discovered, while HLA-B*1502 and HLA-B*5701 were negative. The diagnosis of drugs induced liver injury (DILI) was considered. In general, liver damage would improve once the alleged drugs were withdrawn. As for this patient, the liver parameters did not decline as expected, with AST 48 U/L, ALT 219 U/L, ALP 146 U/L, GGT 1639 U/L, TB 220 μmol/L, and DB 160 μmol/L. As the disease progresses, it may eventually develop into liver failure requiring a liver transplant. Plasma exchange was conducted on December 4 and December 7. Adenosylmethionine, acetylcysteine, ursodeoxycholic acid, and fenofibrate were taken continuously. On December 23, liver biochemical parameters were reevaluated: AST 60 U/L, ALT 77 U/L, ALP 88 U/L, GGT 406 U/L, TB 40.9 μmol/L, and DB 29.8 μmol/L. The patient was discharged home with instructions to continue her ursodeoxycholic acid course. The liver parameters remained normal at several follow-up visits. Without unanticipated events, she resumed the NSCLC regimens precluding dacomitinib, after being evaluated by oncologists.

## 3. Discussion

Liver injury can be induced by many causes, such as hepatophilic viruses, hepatoxic medications or supplements, autoimmunity, and alcohol. In our case, the patient was in her 60s and lived in China; hepatitis B virus infection should be a priority hazard to be excluded. As mentioned above, hepatitis B surface antigen, hepatitis B e antigen, and hepatitis B virus antibodies profile were all negative; antibodies of hepatitis C virus, hepatitis A virus, hepatitis E virus, Epstein–Barr virus antibodies profile, and cytomegalovirus antibodies were negative either; the patient did not present with fever, rash, or lymphadenopathy; the diagnosis of viral hepatitis was not supported. No signs of extrahepatic biliary obstruction were found on ultrasound or magnetic resonance imaging. Immunofluorescence antinucleus antibodies, antismooth muscle antibody, anti-liver/kidney microsome, and antimitochondrial antibody had not been found. The interface hepatitis, emperipolesis, and hepatocyte rosettes usually seen in typical autoimmune hepatitis^[8]^ were not present on the patient liver histology; nonsuppurative cholangitis, fibrosing obliterative cholangitis^[9]^ and concentric “onion skin” periductal fibrosis, bile duct proliferation, and cholangioectasia^[10]^ were not present either; autoimmune liver disease was not supported. There were no neurological complications and the blood ceruloplasmin level was normal; the patient reported no family history of Wilson disease and no alcohol consumption. The diagnosis of DILI was established.

The preferred suspicious culprits for the liver injury were the medications she had taken, including dacomitinib, metoprolol, and loratadine. The diagnosis of DILI was one of the most challenging tasks due to a lack of objective tests. The patient started taking dacomitinib on September 29, 2020, for more than a month; the fatigue was reported before she was prescribed loratadine and metoprolol, though the connectivity between the symptom and dacomitinib had not been proved yet. After 1-month treatment course of dacomitinib, with the loratadine for 3 days and metoprolol for 4 days involved, hepatitis and icterus developed.

The patient visited the clinic for the abnormal liver parameters, with predominant GGT elevation, followed by the progressive increase of TB. The majority of GGT in liver came from biliary epithelial cells and bile canaliculi, suggesting the damage of biliary tracts when elevated. For this patient, the value of GGT reached 2510 U/L and TB reached 233 μmol/L, in which DB was 1.5 times IB, accompanied by the elevation of ALT and AST. The profiles of biochemical parameters suggested the diagnosis of DILI with mixed liver injury. Roussel Uclaf Causality Assessment Method was a validated and widely utilized system for DILI, the score of Roussel Uclaf Causality Assessment Method was 7 in this case (probable adverse drug reaction in the context of this patient’s disease course), R value was 4.57 (mixed liver injury).

### 3.1. Drugs involved in this case

It made DILI diagnosis complicated that several medications had been prescribed to this patient. After taking dacomitinib for about a month, loratadine and metoprolol had been initiated for a short period before her hospitalization.

#### 3.1.1. Dacomitinib.

Dacomitinib was a kind of high-selective, irreversible small molecule inhibitor of EGFR. Solid evidence validated the application in NSCLC with EGFR mutant.^[6,11,12]^ Prior to our study, there were few reports about dacomitinib with conventional dosage inducing hepatotoxity.^[13]^ The common adverse effects included diarrhea, paronychia, dermatitis acneiform, stomatitis, and decreased appetite.^[14]^ There were few reports of liver injury induced by dacomitinib. In recent literature of dacomitinib clinical trials, records of liver injury highlighted that the elevation in serum aminotransferase usually was transient and asymptomatic, which rarely needed to be dose reduction or discontinuation, and there was no report about jaundice as adverse event induced by dacomitinib.^[15,16]^

#### 3.1.2. Loratadine.

Loratadine was a long-acting antihistamine drug with the potential to select the peripheral histamine-1 receptor. It can be metabolized in liver microsomes primarily by the CYP3A4 and CYP2D6, which suggests that the serum concentration of loratadine would not substantially elevate even after CYP2D6 enzyme was inhibited. Schiano et al^[17]^ reported subfulminant liver failure with jaundice, hepatic necrosis, and worsening encephalopathy caused by loratadine 10 mg taken for 14 months. The pathology manifestation showed hepatocyte necrosis, lobular inflammation, proliferating bile ductules with the absence of fibrosis and nodule formation, and also bile plugs were presented with islands of residual hepatocytes. The second case in the same literature took loratadine 10 mg/d for 2 weeks, along with bisoprolol fumarate and hydrochlorothiazide, and liver injury with mild canalicular and intracytoplasmic cholestasis was triggered.

#### 3.1.3. Metoprolol.

Metoprolol, a cardioselective β-adrenoceptor antagonist, was approved for therapy in systemic hypertension, angina pectoris, acute myocardial infarction, arrhythmia cordis, hypertrophic cardiomyopathy, and hyperthyroidism. Instead of liver injury, metoprolol adverse effects preferred the symptom of cardiovascular system to hepatotoxicity. There were a few reports that supported the connection between the metoprolol and liver injury; for instance, metoprolol 100 mg orally per day for about 1 month eventually led to liver failure.^[18]^ For our case, the dosage of metoprolol was 47.5 mg for 4 days. According to the pharmacodynamic characteristics of metoprolol, the plasma protein-binding rate of metoprolol was about 11% to 12%, and the bioavailability was 30% to 40%. The patient did not present with heart failure, bradycardia, atrioventricular block, hypotension, cardiac arrest, respiratory depression, and asphyxia as manifestations of metoprolol overdose. All of these hardly explained the fact that the patient had liver damage for more than a month. It made the assumption that metoprolol should be responsible for liver injury less convincing.^[19]^

### 3.2. The review and the underlying mechanism of the intrahepatic cholestasis

To further investigate the association of cholestasis with dacomitinib, we conducted the review of clinical trials and public literature with the keyword of “dacomitinib” and “drug induced liver injury”, “DILI”, “cholestasis”, “intra-hepatic cholestasis”, “hepatotoxicity”, “icterus”, and “jaundice”. Wu et al^[13]^ reported EGFR-TKI-induced hepatotoxicity. A phase II clinical trial (ClinicalTrials.gov identifier: NCT01858389)^[20]^ reported that liver injury adverse events associated with dacomitinib were mostly mild transaminase elevation in 38 patients with advanced NSCLCs, except for 1 case of grade 3 (3–10 upper limit of normal according to Common Terminology Criteria for Adverse Events) hyperbilirubinemia. In another study with a larger sample, dacomitinib (PF-00299804) vs erlotinib in the treatment of advanced NSCLC (ARCHER 1009, ClinicalTrials.gov identifier: NCT01360554)^[21]^ reported that 8 of 436 (1.83%) cases presented with mild and moderate ALT increased, 10 of 436 (2.29%) cases with mild and moderate AST increased in dacomitinib group, and there was 1 case with hyperbilirubinemia. Similar findings had been reported in other studies,^[6]^ such as NCT01465802, NCT00728468, and NCT00548093.^[11]^ A phase II, open-label trial of dacomitinib (PF-00299804), as a single oral agent in selected patients with adenocarcinoma of the lung (ClinicalTrials.gov identifier: NCT00818441),^[22]^ reported a case death, who developed hepatic failure likely due to an interaction of dacomitinib and mirtazapine, which metabolized by CYP2D6. Sugiyama et al^[23]^ reported that single-nucleotide polymorphisms in metabolic enzymes played roles in severe hepatotoxicity induced by EGFR-TKI gefitinib; among 9 patients who developed severe liver injury, 8 patients were confirmed with poor metabolic (PM) phenotypes of metabolic enzymes for CYP2D6 or CYP3A5. The result found that the incidence rate of severe hepatotoxicity was significantly higher among patients with PM phenotypes than those without. Reckamp et al^[11]^ reported 8 cases of liver injury in a phase 2 trial of dacomitinib in patients with advanced NSCLC after failure of prior chemotherapy and erlotinib. In summary, the risk of dacomitinib causing severe cholestasis was low and could be relevant to specific population.

The mechanism of liver injury induced by dacomitinib remained unclear, variant capacity of liver metabolic enzymes accounted partly for the frequency of hepatotoxicity.

#### 3.2.1. CYP2D6 and dacomitinib.

CYP2D6 was involved in the metabolism of approximately 20% of currently approved drugs.^[24]^ Based on the metabolic enzyme activity corresponding to different genotypes, the population was divided into 4 types of metabolic capacity, including ultrarapid metabolic, normal metabolic, intermediate metabolic (IM), and PM.^[25]^ There were 74 allelic variants, and a series of subvariants of the CYP2D6 gene had been identified and the number continued to grow.^[26]^ Dacomitinib was a substrate for CYP2D6; also, it was an inhibitor of CYP2D6. The CYP2D6*10/*10 had been confirmed by the gene test of the liver tissue in this case. It was one of the IMs that reduced metabolic enzyme activity.^[27]^ Pharmacokinetically, in healthy Chinese subjects, dacomitinib achieved steady blood concentrations after 14 days of dosing, and its terminal half-life was 62.66 hours^[28]^; it was necessary to take 2 to 3 weeks for its metabolites to be completely cleared from the body. Meanwhile, metoprolol was primarily metabolized through the CYP2D6 pathway, which competitively inhibits dacomitinib metabolism.

#### 3.2.2. ABCB1 GG variants and dacomitinib.

ABCB1, localized at the canalicular membrane of hepatocytes, was related to hepatic bile excretion. It was perceived to be contributing to the canalicular excretion of drugs and other xeno chemicals into bile. In accordance with database of single-nucleotide polymorphisms, minor allele frequency of ABCB1 GG variants (rs1045642) was 0.243, and database search of PharmGKB did not identify any adverse interactions between the ABCB1 GG genotype and the 3 drugs mentioned above. Nevertheless, dacomitinib can downregulate the ABCB1 and ATP-binding cassette subfamily G member 2 to antagonize the efflux function of hepatocytes,^[29,30]^ increasing the risk of intrahepatic cholestasis; while dacomitinib itself was mainly metabolized in the liver, 79% was excreted in the feces, of which 20% was excreted in the prototype form.

In summary, for the patient with IMs of CYP2D6, dacomitinib was competitively inhibited by metoprolol when metabolized simultaneously. In addition, the serum protein-binding rate of dacomitinib was nearly 98%, and half-life elimination was almost 70 hours. Dacomitinib impaired bile excretion by downregulating ABCB1 and ATP-binding cassette subfamily G member 2 expression. All of these factors led to the persistent accumulation of dacomitinib blood concentrations and made it difficult for the intrahepatic cholestasis to recover in the short term. Taken together, the refractory cholestasis was an inevitable outcome.

After admission, the patient received the conventional treatment for nearly a month, but the serum bilirubin and GGT remained at a pronounced high level. To remove dacomitinib from the body as soon as possible, plasma exchange was performed on December 4 and December 7, and intrahepatic cholestasis was under control and gradually improving. On December 23, liver parameters were rechecked, indicating significant improvement in the serum levels of bilirubin and GGT. The patient had learned to pay the regular follow-up visits to monitor the potential liver damage while in the NSCLC regimens.

In conclusion, our case highlighted that severe intrahepatic cholestasis associated with dacomitinib hepatotoxicity warranted more awareness, and drug interactions should be monitored, especially liver metabolic enzymes CYP2D6 were involved.

## Author Contributions

All the authors carried out the treatment, Fei Qiao and Qinlei Chen: Writing-Original draft preparation, Nanyuan Fang: Data curation and Writing-Reviewing and Editing, Weiting Lu: Liver pathology report.
